# Role of Synaptophysin, Chromogranin and CD56 in adenocarcinoma and squamous cell carcinoma of the lung lacking morphological features of neuroendocrine differentiation: a retrospective large-scale study on 1170 tissue samples

**DOI:** 10.1186/s12885-021-08140-9

**Published:** 2021-05-01

**Authors:** Katharina Kriegsmann, Christiane Zgorzelski, Thomas Muley, Petros Christopoulos, Michael Thomas, Hauke Winter, Martin Eichhorn, Florian Eichhorn, Moritz von Winterfeld, Esther Herpel, Benjamin Goeppert, Albrecht Stenzinger, Felix J. F. Herth, Arne Warth, Mark Kriegsmann

**Affiliations:** 1 Department of Hematology, Oncology and Rheumatology, University Hospital Heidelberg, Heidelberg, Germany; 2 Institute of Pathology, University Hospital Heidelberg, Im Neuenheimer Feld 224, Heidelberg, Germany; 3 Translational Lung Research Centre Heidelberg, Member of the German Centre for Lung Research (DZL), Heidelberg, Germany; 4 Translational Research Unit, Thoraxklinik at Heidelberg University, Heidelberg, Germany; 5 Department of Thoracic Oncology, Thoraxklinik at Heidelberg University, Heidelberg, Germany; 6 Department of Thoracic Surgery, Thoraxklinik at Heidelberg University, Heidelberg, Germany; 7 Department of Pneumology and Critical Care Medicine, Thoraxklinik at Heidelberg University, Heidelberg, Germany; 8 Institute of Pathology, Cytopathology, and Molecular Pathology, UEGP MVZ, Gießen, Wetzlar, Limburg Germany

**Keywords:** Synaptophysin, Chromogranin, CD56, Immunohistochemistry, Non-small cell lung cancer

## Abstract

**Background:**

Synaptophysin, chromogranin and CD56 are recommended markers to identify pulmonary tumors with neuroendocrine differentiation. Whether the expression of these markers in pulmonary adenocarcinoma and pulmonary squamous cell carcinoma is a prognostic factor has been a matter of debate. Therefore, we investigated retrospectively a large cohort to expand the data on the role of synaptophysin, chromogranin and CD56 in non-small cell lung cancer lacking morphological features of neuroendocrine differentiation.

**Methods:**

A cohort of 627 pulmonary adenocarcinomas (ADC) and 543 squamous cell carcinomas (SqCC) lacking morphological features of neuroendocrine differentiation was assembled and a tissue microarray was constructed. All cases were stained with synaptophysin, chromogranin and CD56. Positivity was defined as > 1% positive tumor cells. Data was correlated with clinico-pathological features including overall and disease free survival.

**Results:**

110 (18%) ADC and 80 (15%) SqCC were positive for either synaptophysin, chromogranin, CD56 or a combination. The most commonly positive single marker was synaptophysin. The least common positive marker was chromogranin. A combination of ≤2 neuroendocrine markers was positive in 2–3% of ADC and 0–1% of SqCC. There was no significant difference in overall survival in tumors with positivity for neuroendocrine markers neither in ADC (univariate: *P* = 0.4; hazard ratio [HR] = 0.867; multivariate: *P* = 0.5; HR = 0.876) nor in SqCC (univariate: *P* = 0.1; HR = 0.694; multivariate: P = 0.1, HR = 0.697). Likewise, there was no significant difference in disease free survival.

**Conclusions:**

We report on a cohort of 1170 cases that synaptophysin, chromogranin and CD56 are commonly expressed in ADC and SqCC and that their expression has no impact on survival, supporting the current best practice guidelines.

## Background

Synaptophysin, chromogranin and CD56 are recommended markers to identify pulmonary tumors with neuroendocrine differentiation [1]. These markers are frequently used to confirm a diagnosis of typical carcinoid, atypical carcinoid, small cell lung cancer and large cell neuroendocrine carcinoma (LCNEC). In the routine diagnostic setting, particularly the differentiation of LCNEC and pulmonary adenocarcinoma (ADC) with solid growth pattern or non-keratinizing squamous cell carcinoma (SqCC) might be challenging. According to current guidelines only non-small cell carcinomas (NSCLC) that exhibit morphological features of neuroendocrine differentiation should be stained with neuroendocrine markers. In case of a negative result these tumors should be labelled NSCLC with neuroendocrine morphology in biopsy specimens with a comment that the tumor is suspected to exhibit neuroendocrine differentiation that could not be confirmed by immunobiological staining. On the other hand, ADC and SqCC may show the expression of neuroendocrine markers despite the lack of neuroendocrine morphology. The clinical significance in this constellation has been investigated in previous studies [2–11]. While some of the studies suggested an impact of neuroendocrine marker expression on survival [4, 7, 12–16] most of the studies reported no prediction of survival [2, 10, 11]. In this study we investigated over 1000 patient samples to expand the data on the role of synaptophysin, chromogranin and CD56 in NSCLC lacking morphological features of neuroendocrine differentiation.

## Methods

### Patient cohort

Formalin fixed and paraffin embedded NSCLC specimens resected from 2002 to 2010 in the Thoracic Hospital Heidelberg at Heidelberg University were extracted from the archive of the Institute of Pathology, Heidelberg University, with the support of the tissue bank of the National Center for Tumour Diseases. Tissues were used in accordance with the ethical regulations of the NCT tissue bank defined by the local ethics committee (#S315–2020, NCT#2603). Diagnoses were made according to the recommendations of the 2015 world health classification of tumours of the lung, thymus and heart [1]. One thousand one hundred seventy patients with NSCLC including ADC and SqCC were selected. Tissue microarrays were constructed as described previously [17, 18].

### Immunohistochemistry

**Fig. 1:**
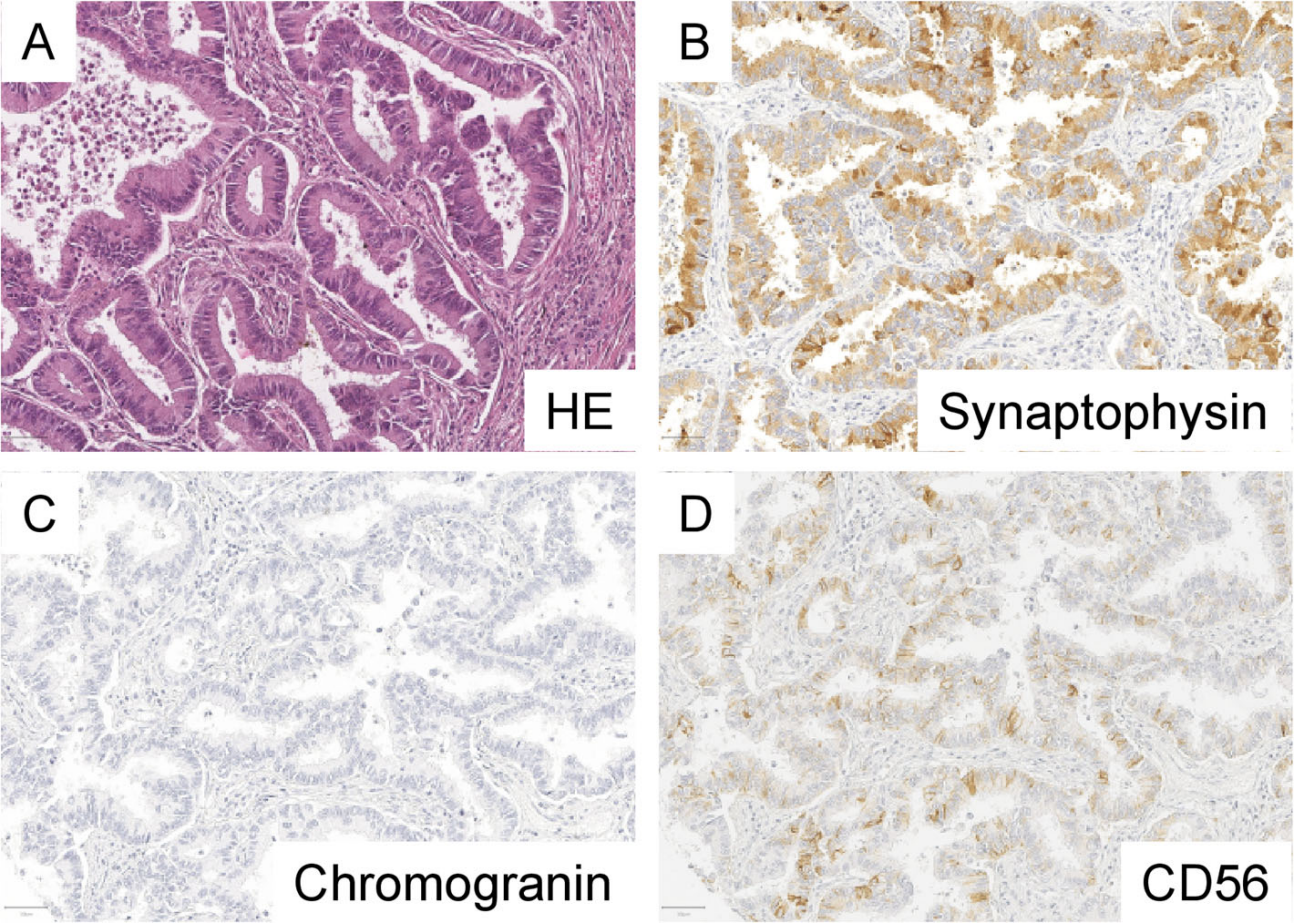
Example of a pulmonary adenocarcinoma positive for neuroendocrine markers. The typical acinar growth pattern of pulmonary adenocarcinoma is seen (**a**, HE, 200x). Synaptophysin shows homogenous moderate to strong positivity (**b**, Synaptophysin, 200x). Chromogranin is negative (**c**, Chromogranin, 200x). CD56 shows focal moderate positivity (**d**, CD56, 200x)

**Fig. 2:**
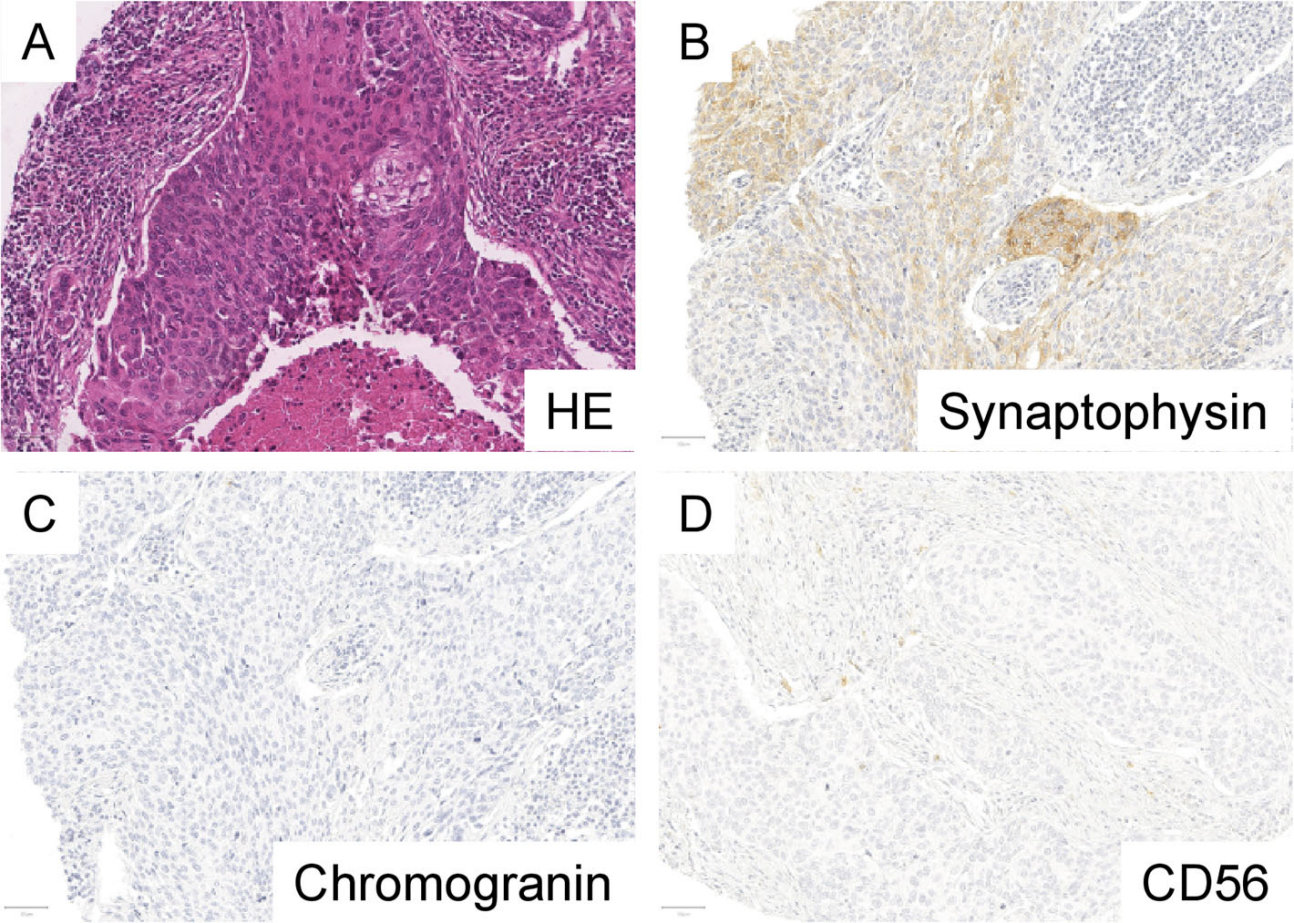
Example of a pulmonary squamous cell carcinoma positive for neuroendocrine markers. Typical morphological features of squamous cell carcinoma with local dyskeratosis is seen (**a**, HE, 200x). Synaptophysin shows focal moderate positivity (**b**, Synaptophysin, 200x). Chromogranin and CD56 are negative in this example (**c**, Chromogranin, **d**, CD56, 200x)

Immunohistochemical (IHC) staining was performed as previously described [18, 19]. In brief, slides were deparaffinized, pretreated with an antigen retrieval buffer and stained using an automated device. Immunohistochemical stainings were performed on a Ventana Benchmark Ultra (Roche, Switzerland). The antibody and staining conditions are shown in Table 1. The evaluation was carried out by an experienced pathologist (MK). Synaptophysin and chromogranin were considered when located in the cytoplasm, CD56 was evaluated when located on the membrane. Positivity of a marker was defined as > 1% positive tumor cells, as in previous studies [2]. Typical examples of positive and negative staining results of ADC and SqCC are shown in Figs. 1 and 2. The results from the conventional NSCLC markers TTF-1 and p40 were published previously [20, 21].

**Table 1:** Antibodies used and staining conditions

Antibody	Company	Clone	Pretreatment	Buffer incubation time (min)	Antibody incubation time (min)	Dilution
p40	Ventana	BC28	Tris/Borat/ EDTA, pH 8.4	48	24	RTU
TTF-1	Novocastra	SPT24	Tris/Borat/ EDTA, pH 8.4	56	24	1:100
Synaptophysin	Cell Marque	MRQ-40	Tris/Borat/ EDTA, pH 8.4	48	24	RTU
Chromogranin A	Dako	polyclonal	Tris/Borat/ EDTA, pH 8.4	32	24	1:400
CD56	Ventana	MRQ-42	Tris/Borat/ EDTA, pH 8.4	40	24	RTU

*CD* cluster of differentiation, *TTF-1* thyroid transcription factor 1

### Molecular data

Molecular data included results for KRAS, EGFR, BRAF, ROS1 and ALK testing were available for ADC from a previous investigation [22]. In brief, cases were analyzed by Sanger sequencing for *KRAS* (exon 1), *EGFR* (exons 18–21) and *BRAF* (exon 15). Cases tested for *ROS1* and *ALK* were prescreen using IHC, results were subsequently validated by fluorescence in situ hybridization (FISH) using a break-apart probe. Only cases with FISH-confirmation were considered positive.

### Data analysis

Statistical analyses were performed using R-Statistical Software (www.r-project.org, v.4.0.0, Free Software Foundation), R-Studio (v. 1.2.5042, Affero General Public License, USA), or Excel 2019 (Microsoft, USA). Correlation of the immunohistochemical stains with clinicopathological characteristics was by the unpaired t-test for numerical and by the Fisher-Freeman-Halton test for categorical variables. Analysis of overall survival (OS), disease-free survival (DFS) and Kaplan-Meier plots were done with the survival and the survminer package in R. In the multivariate Cox regression model no model selection procedures were applied as we aimed to fit a model with all, from the clinical/diagnostic point of few, main effects and also show the missing impact of statistically not significant variables. *P*-values < 0.05 were considered significant.

## Results

### Patient characteristics

Overall, 1170 NSCLC including 627 ADC and 543 SqCC were analyzed. 816 (70%) patients were male, 354 (30%) were female. Median age was 64 years (min-max: 30–89 years). Most patients underwent surgery with pT2 tumors and negative lymph-node status.

### Expression of p40, TTF-1, Synaptophysin, Chromogranin and CD56

548 (87%) ADC were positive with antibodies against TTF-1. Only 8 (1%) ADC showed positivity against p40. These cases also exhibited positivity for TTF-1 in the same tumor cells and showed a typical growth pattern of adenocarcinoma. The vast majority of ADC were negative for p40 (99%). 511 (94%) SqCC were positive with antibodies against p40. Only 6 (1%) SqCC exhibited focal weak TTF-1 positivity. These tumors showed keratinization and intercellular bridges and were therefore classified as SqCC. The majority of SqCC were negative for TTF-1 (99%). None of the ADC and SqCC showed morphological features of neuroendocrine differentiation.

**Fig. 3:**
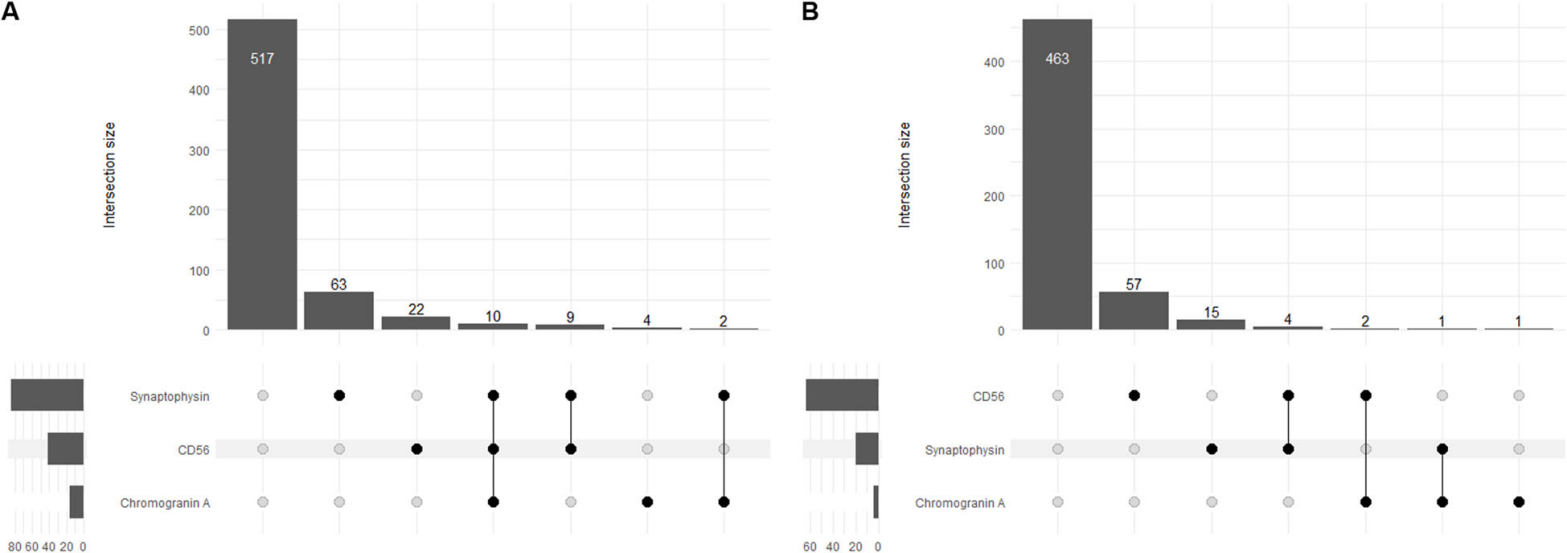
Upset plots indicating the proportion of neuroendocrine marker positivity in ADC and SqCC. **a** ADC, adenocarcinoma; **b** SqCC, squamous cell carcinoma

Overall, 110 (18%) ADC and 80 (15%) SqCC were positive for either synaptophysin, chromogranin, CD56 or a combination of these. The most commonly positive single marker was synaptophysin in ADC (13%) and SqCC (4%). The least common positive marker was chromogranin in ADC (3%) and CD56 in SqCC (1%). A combination of either two or three neuroendocrine markers was positive in 2–3% of ADC and 0–1% of SqCC. A summary of the expression of p40, TTF-1 and the neuroendocrine markers is provided in Table 2 and Fig. 3. No significant difference of gender, age, T- and N-categories as well as clinical stage were observed between ADC and SqCC with and without expression of neuroendocrine markers (Tables 3 and 4).

**Table 2:** IHC staining characteristics of ADC and SqCC tumors

	ADC		SqCC	
	n	%	n	%
**Patients, n**	627	100	543	100
**General NSCLC markers**				
TTF1				
Positivity	548	87	6	1
Negativity	79	13	537	99
p40				
Positivity	8	1	511	94
Negativity	619	99	32	6
**Positivity for neuroendocrine marker**				
Overall^a^	110	18	80	15
Synaptophysin	84	13	20	4
Chromogranin A	16	3	4	1
CD56	41	7	3	1
Synaptophysin / Chromogranin A	12	2	1	0
Synaptophysin / CD56	19	3	4	1
Chromogranin A / CD56	10	2	2	0
Synaptophysin / Chromogranin A / CD56	10	2	0	0

*ADC* adenocarcinoma, *IHC* immunohistochemistry, *NSCLC* non-small cell lung carcinoma, *SqCC* squamous cell carcinoma

^a^Overall positivity was defined as positivity for ≥1 neuroendocrine marker

**Table 3:** ADC patient characteristics and stratification by neuroendocrine marker

	ADC overall cohort		ADC neuroendocrine marker positive		ADC neuroendocrine marker negative		***p*** value
	n	%	n	%	n	%	
**Patients**	627	100	110	100	517	100	
**Gender**							
Male	365	58	67	61	298	58	0.528
Female	262	42	43	39	219	42	
**Age, median y (range)**	63 (30–89)		63 (41–84)		63 (30–89)		0.373
**TNM**							
pT							
pT1	127	20	25	23	102	20	0.535
pT2	388	62	63	57	325	63	
pT3	94	15	17	15	77	15	
pT4	18	3	5	5	13	3	
pN							
pN0	314	50	63	57	251	49	0.068^a^
pN1	94	15	14	13	80	15	
pN2	192	31	28	25	164	32	
pN3	5	1	0	0	5	1	
pNX	22	4	5	5	17	3	
pM							
pM1	26	4	2	2	24	5	
pMX	601	96	108	98	493	95	
**Stage**							
I	254	41	46	42	208	40	0.153^b^
II	130	21	29	26	101	20	
III	217	35	33	30	184	36	
IV	26	4	2	2	24	5	
**Genetic aberrations**							
KRAS	147^c^	36	29^d^	36	118^e^	36	0.732^f^
EGFR	64^c^	16	10^d^	8	52	16	
BRAF	14^c^	3	2^d^	3	12	4	
ROS1	5^c^	1	1^d^	1	4	1	
ALK	5^c^	1	0^d^	0	5	2	

*ADC* adenocarcinoma, *M* metastases, *N* nodal stage, *T* tumor size, *y* year

^a^pN0 versus pN1/pN2/pN3; pNX not included

^b^stage I versus II versus III/IV

^c^available for 405 cases

^d^available for 80 cases

^e^available for 327 cases

^f^KRAS versus EGFR versus BRAF/ROS1/ALK

**Table 4:** SqCC patient characteristics and stratification by neuroendocrine marker

	SqCC overall cohort		SqCC neuroendocrine marker positive		SqCC neuroendocrine marker negative		***p*** value
	n	%	n	%	n	%	
**Patients**	543	100	80	100	463		
**Gender**							
Male	451	83	67	84	384	83	0.858
Female	92	17	13	16	79	17	
**Age, median y (range)**	65 (38–83)		64 (40–82)		65 (38–83)		0.428
**TNM**							
pT							
pT1	106	20	16	20	90	19	0.645
pT2	324	60	48	60	276	60	
pT3	93	17	15	19	78	17	
pT4	20	4	1	1	19	4	
pN							
pN0	255	47	35	44	220	48	0.570
pN1	179	33	23	29	156	34	
pN2	98	18	20	25	78	17	
pN3	1	0	0	0	1	0	
pNX	10	2	2	3	8	2	
pM							
pM1	8	1	2	3	6	1	
pMX	535	99	78	98	457	99	
**Stage**							
I	185	34	28	35	157	34	0.437
II	208	38	26	33	182	39	
III	142	26	24	30	118	25	
IV	8	1	2	3	6	1	

*M* metastases, *N* nodal stage, *SqCC* squamous cell carcinoma, *T* tumor size, *y* year

^a^pN0 versus pN1/pN2/pN3; pNX not included

^b^stage I versus II versus III/IV

### Survival analysis

**Fig. 4:**
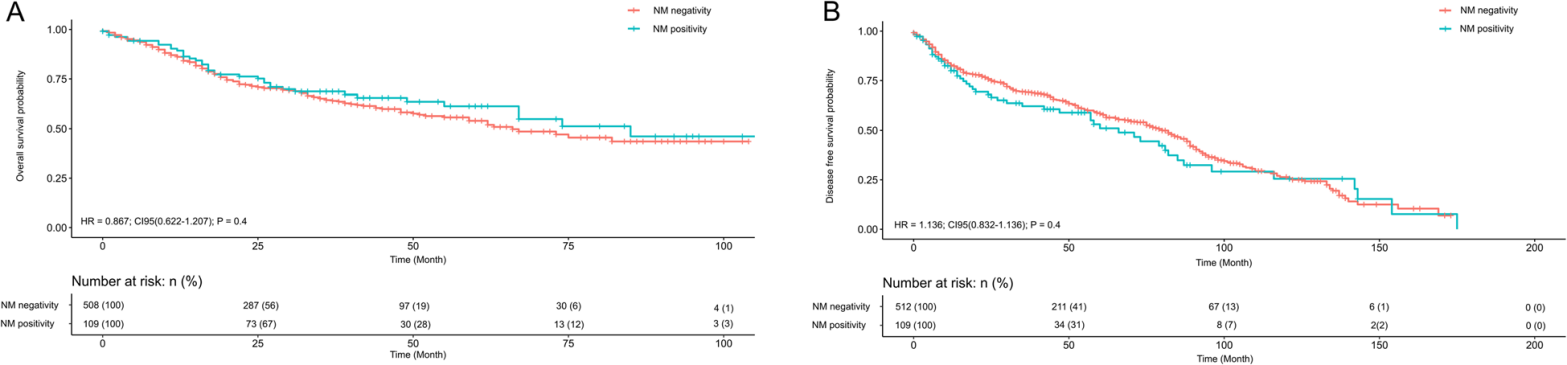
Univariate OS and DFS analysis of ADC cases with regard to positivity and negativity of neuroendocrine marker. Overall positivity was defined as positivity for ≥1 neuroendocrine marker. NM, neuroendocrine marker; OS, overall survival

**Fig. 5:**
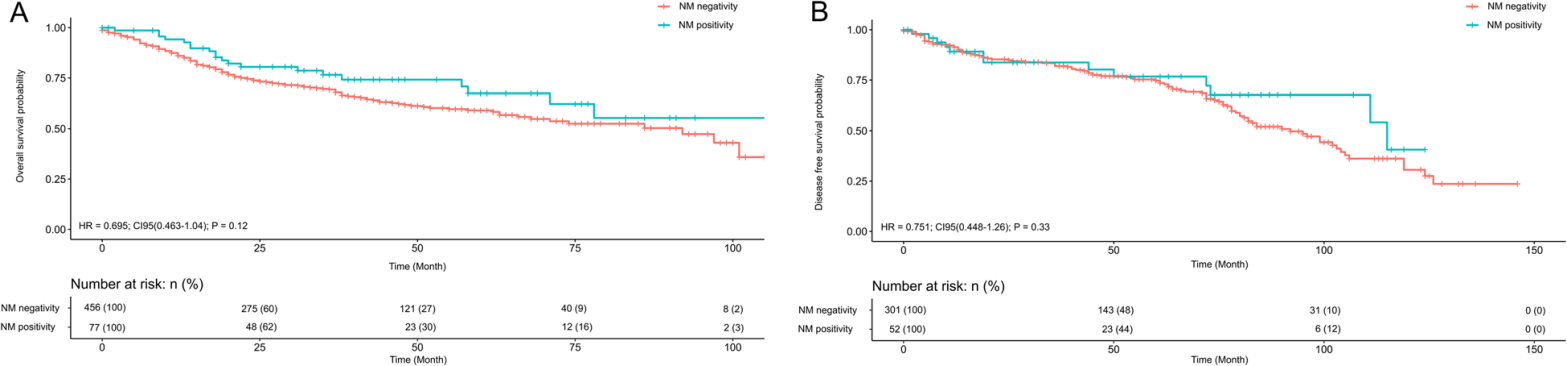
Univariate OS and DFS analysis of SqCC cases with regard to positivity and negativity of neuroendocrine marker. Overall positivity was defined as positivity for ≥1 neuroendocrine marker. NM, neuroendocrine marker; OS, overall survival

OS was analyzed in patients with ADC and SqCC with respect to the expression of neuroendocrine markers. Although survival in ADC and SqCC with expression of neuroendocrine marker expression was better, but no significant difference was detected in univariate OS analysis in ADC (*P* = 0.4; hazard ratio [HR] = 0.867; 95% confidence interval [CI95 = 0.622–1.207]) and SqCC (*P* = 0.1; HR = 0.694 [CI95 = 0.462–1.042]. Likewise, no significant difference was detected in univariate DFS in ADC (P = 0.4; HR = 1.136; CI95 = 0.832–1.136) and SqCC (*P* = 0.3; CI95 = 0.448–1.260). Kaplan-Meier plots are shown in Figs. 4 and 5.

Multivariate Cox-proportional hazard analysis for OS showed a significant impact of clinical stage and gender in ADC, but only of clinical stage in SqCC. No significance of neuroendocrine marker expression was detected for ADC and SqCC regarding OS in multivariate analysis (Tables 5 and 6).

**Table 5:** Multivariate Cox proportional hazard analysis for OS in ADC

Variable	HR (CI_**95**_)	***p*** value
Stage II	2.76 (1.576–3.581)	**< 0.001**^**a**^
Stage III	4.649 (3.276–6.597)	**< 0.001**^**a**^
Stage IV	6.729 (3.726–12.155)	**< 0.001**^**a**^
Age (> 59 versus < 59 years)	1.036 (0.776–1.384)	0.809
Gender (female versus male)	0.564 (0.420–0.757)	**< 0.001**
Neuroendocrine marker (positivity versus negativity)	0.876 (0.616–1.247)	0.463

*n* = 617

^a^as compared to Stage I

*OS* overall survival

**Table 6:** Multivariate Cox proportional hazard analysis for OS in SqCC

Variable	HR (CI_**95**_)	***p*** value
Stage II	1.657 (1.135–2.419)	**0.009**^**a**^
Stage III	2.889 (1.954–4.274)	**< 0.001**^**a**^
Stage IV	4.205 (1.298–13.624)	**0.017**^**a**^
Age (> 59 versus < 59 years)	1.282 (0.900–1.826)	0.168
Gender (female versus male)	0.790 (0.504–1.239)	0.305
Neuroendocrine marker (positivity versus negativity)	0.697 (0.436–1.113)	0.131

*n* = 533

*OS* overall survival

^a^as compared to Stage I

## Discussion

In the present study we investigated the impact of the expression of synaptophysin, chromogranin and CD56 in ADC and SqCC without neuroendocrine morphology on overall survival in a large study including more than 1000 patients. This is the largest cohort reported on this topic to date. We found that neuroendocrine marker expression is common and is not associated with OS and DFS.

Staining a combination of synaptophysin, chromogranin and CD56 is currently advised to establish evidence of neuroendocrine differentiation in thoracic tumors [23]. However, staining should be restricted to NSCLC exhibiting neuroendocrine differentiation, as it has been shown that ADC and SqCC may exhibit positive staining in 10–30% in most studies [2, 6]. Studies reporting a higher positivity rate were commonly done on whole slides [3] and not on tissue micro-arrays [2, 4, 11], with one exception reporting neuroendocrine marker expression in up to 90% of tumors [5]. Thus, our results are in line with the literature [3, 6, 10]. The differences of the reported positivity rates might also be explained by different cut-offs for the definition of positivity and the application of different antibody clones [5, 10, 11]. We investigated only one cut-off value for positivity and choose a cut-off of > 1% positive tumor cells. This cut-off has been used in other previous studies but is somewhat arbitrary [2, 3]. We decided to use this cut-off as single cell positivity is a physiologic finding in lung tissue and single neuroendocrine cells overgrown by tumor cells and unspecific background staining might not be reliably distinguished from positive tumor cells [24]. Moreover, cut-off values above 1% are rarely helpful in the routine diagnostic setting. Ionescu et al. reported CD56 to be most commonly expressed closely followed by synaptophysin [2], while Sterlacci et al. reported synaptophysin to be the most commonly detected positive marker in ADC and SqCC, as in our study [11]. In line with these large-scale investigations, chromogranin was least commonly expressed in our study.

 The impact of neuroendocrine marker expression on survival of patients with ADC and SqCC is controversially discussed. While most investigations found no impact on prognosis, some more recent studies challenged this finding [4, 25, 26]. Feng et al. investigated the impact of neuroendocrine marker expression on OS and DFS in one of the largest cohorts including a total of 451 patients and found a significantly worse survival in patients with tumors expressing neuroendocrine markers [4]. However, another large study including more than 200 ADC and SqCC did not find any prognostic impact neither on OS nor DFS, in line with the findings of our study [2].

Another marker of neuroendocrine differentiation, Insulinoma-associated Protein 1 (INSM1), has been reported to support the diagnosis of neuroendocrine differentiation in thoracic tumors and has the potential to complement the currently recommended neuroendocrine markers [27, 28]. Interestingly, INSM1 has been reported to be more sensitive and specific as compared to the single markers Synaptophysin, Chromogranin and CD56 and was therefore advocated as a first-line stand alone marker or in combination with CD56 to detect neuroendocrine differentiation [28–31]. INSM1 marker expression has been suggested to be prognostic in high-grade neuroendocrine neoplasms, but if INSM1 expression has a prognostic impact in ADC or SqCC remains to be investigated [32].

Moreover, we could not detect any differences in the rate of common genetic aberrations in pulmonary ADC when we compared tumors with and without expression of neuroendocrine markers. Although we analyzed a large cohort, these data must be interpreted with caution, because the respective patient subsets were small.

Our study has several limitations: first, the retrospective design of the investigation. Prospective large-scale studies are not available to the best of our knowledge. Second, we used a tissue microarray as a surrogate for the biopsy situation. As only two cores from the whole tumor were investigated, it is not entirely clear if other parts of tumors that were judged negative in our study exhibit neuroendocrine immunoreactivity. This problem is also highlighted by the fact that previous studies on whole slides reported higher rates of neuroendocrine positivity [3]. On the other hand numerous studies comparing the results of tissue microarray studies with the findings from conventional large sections using other biomarkers have shown that all well-established associations between molecular markers and tumor phenotype or patient prognosis can be reproduced with tissue microarrays [33].

## Conclusion

In summary, we show that synaptophysin, chromogranin and CD56 are commonly expressed in ADC and SqCC and that their expression as no impact on OS and DFS supporting the current best practice guidelines.

## Data Availability

The datasets used and/or analyzed during the current study are available from the corresponding author on reasonable request.

## Abbreviations

ADC: Adenocarcinoma
CD: Cluster of differentiation
DFS: Disease-free survival
FISH: Fluorescence in situ hybridization
IHC: Immunohistochemistry
INSM1: Insulinoma-associated Protein 1
LCNEC: Large cell neuroendocrine carcinoma
NSCLC: Non-small cell lung cancer
OS: Overall survival
SqCC: Squamous cell carcinoma
